# Prediction of success in sports based on assumed individual genetic predisposition: lack of association with the C > T variant in the *ACTN3* gene

**DOI:** 10.1186/s40101-025-00386-7

**Published:** 2025-02-14

**Authors:** Elena Godina, Nikita Khromov-Borisov, Elvira Bondareva

**Affiliations:** 1 Anuchin Research Institute and Museum of Anthropology, Lomonosov Moscow State University, Mokhovaya St., 11, Moscow, 125009 Russia; 2 Russian University of Sports (GTSOLIFK), Syrenevy Blvd., 4. Moscow 105122, Moscow, Russia; 3 Commission On Pseudoscience of Russian Academy of Sciences, Leninsky Prospect, Moscow, 119991 Russia; 4 Lopukhin Federal Research and Clinical Center of Physical-Chemical Medicine of Federal Medical Biological Agency, Malaya Pirogovskaya St., 1a, Moscow, 119435 Russia

**Keywords:** Sport genetics, ACTN3, Gene, Sport, Genetic polymorphism, SNV, Meta-analysis, Genetic testing

## Abstract

**Background:**

Prediction of sports success (sports talent) based on individual genetic characteristics is the main goal of sports genetics/genomics. Most often, markers of predisposition to speed-strength sports, or endurance, are single-nucleotide variants in various parts of DNA. One of the most studied variants is the C/T variant in the ACTN3 gene. The accumulated data on the association of this variant with success in various sports is sufficient to conduct a meta-analysis. The purpose of the present review is to analyze the prognostic utility of the data presented in the literature on molecular genetic markers of genetic predisposition to achieve outstanding sports results using the example of the C > T variant *of ACTN3 (rs1815739).*

**Main body:**

A total of 42 studies were included in the analysis, with a total number of 41,054 individuals (of which 10,442 were in the athlete group and 30,612 in the control group). For each study included in the analysis, the agreement of genotype frequencies with Hardy–Weinberg equilibrium was tested, as well as the presence of an excess or deficit of heterozygotes. Prediction intervals for the overall effect size (OR—odds ratio) was estimated. Both in the subgroups of athletes and controls, a significant difference *F*_IS_ from zero was found, suggesting inbreeding or outbreeding, as well as a very wide 95% CI for *F*_IS_. A meta-analysis was conducted for dominant, codominant, and recessive inheritance models. The obtained ORs and their 95% CIs were in the range of almost negligible values or have very wide CIs. The evaluation for the recessive model showed 95% *PI* for the *OR* lies between 0.74 to 1.92. Statistically, it does not differ from zero, which means that in some 95% of studies comparable to those in the analysis, the true effect size will fall in this interval.

**Conclusion:**

Despite numerous attempts to identify genetic variants associated with success in elite sports, progress in this direction remains insignificant. Thus, no sports or sports roles were found for which the C > T variant of the *ACTN3* gene would be a reliable prognostic marker for assessing an individual predisposition to achieve high sports performance. The results of the present meta-analysis support the conclusion that neutral gene polymorphism—from evolutionary or adaptive point of view—is not a trait that can be selected or used as a predictive tool in sports.

## Background

Over the past decades, numerous attempts have been made to find genes that determine various morphofunctional and psychophysiological characteristics associated with high athletic performances [1, 2]. The first scientific publications devoted to the genetic determinants of athletic success were the works by H. Montgomery et al. and Rivera M.A. et al. [3, 4]. The first monograph on the genetic basis of physical activity, “Genetics of Fitness and Physical Performance,” was published in 1997 by Claude Bouchard et al. [5]. Advances in genotyping technologies have made it possible to identify various gene variants (single-nucleotide substitutions, insertions, and deletions) that can directly or indirectly affect physical performance. Case–control association studies are based on a comparison of allele/genotype frequencies in specific genes or large regions of the genome in athletes and non-athletes. The results of these studies revealed some genes associated with the qualification of the athletes or with their physiological characteristics. Several editions of genetic maps have been published to demonstrate association or linkage with athlete’s phenotype [6]. The most studied in sports genetics are insertion-deletion variants (indel) in the angiotensin-I-converting enzyme gene (*ACE* I > D, rs1799752) and single-nucleotide substitution in the alpha-actinin 3 gene (*ACTN3* C > T). Variants of C > T base substitution in the alpha-actinin 3 gene (*ACTN3*, rs1815739) have been shown to be associated with speed-strength qualities of skeletal muscles. This gene is expressed only in rapidly contracting glycolytic fibers, and the combination of two T alleles (nonsense alleles) leads to a complete absence of ACTN3. Yang and colleagues showed for the first time that the TT genotype is less common in the group of highly qualified sprinters compared to non-athletes and long-distance runners. In the group of highly qualified athletes engaged in strength sports, this genotype was not found at all [7].

This result has been replicated in several other independent studies [8, 9], but other studies have provided conflicting data [10]. The contribution of *ACTN3* to the development of speed-strength qualities is estimated at 1–3% [11]. Even the results of the association of C*ACTN3 with sprint predisposition, which are reproduced in several independent studies, have low specificity and sensitivity. Therefore, it is not clear how this trait—the carrier of the *CC*, *CT*, or *TT* genotype—can help to select for specific sports. In the European population, the frequency of the *C*-allele reaches 80% [7], in some African populations—99% [12]. A study of elite sprinters in Jamaica and the United States (the group of athletes with the best 100 m run results on record, Olympic champions and world record holders) found no significant differences in the frequencies of *ACTN3* genotypes between the athletes and the controls. Ninety-seven percent of those examined in the control group had at least one C-allele [10]. Due to the fact that C > T base substitution is extremely common in the world’s populations and the frequency of the C-allele is high, it is not possible to use genotype data at the individual level. These assumptions are supported by the exceptions revealed in some studies. Thus, highly qualified sprinters (100 m run)—a man and a woman—who passed the qualification for the Olympic Games, were carriers of the TT*ACTN3 genotype [11]. In another study, an athlete who won a silver medal at the 2012 Olympic Games in the long jump, a sport that requires high speed, strength, and explosive qualities of skeletal muscles, was also found to have a *TT* genotype [13]. The very concept of an “elite” athlete does not have a clear definition [14], so it can be difficult to compare various case–control studies in which a group of “elite athletes” is opposed to a control group [15]. The lack of a clear phenotypic (anthropometric, physiological, ethnic, etc.) characteristic of the examined groups of athletes is one of the bottlenecks of sports genetics. The lack of a clearly defined phenotype, which should be inherent in an athlete of the highest level, stimulates the development of a new research direction at the intersection of sports physiology, psychology, anthropology, and genetics—*the phenomics*. Its goal is to accumulate and analyze multivariate data on various characteristics of athletes at the organismal level [16].

The inclusion of people who do not have significant sports results at the time of the study to the group of non-athletes (control) also causes justified criticism [17]. Often, such a group consists of volunteers who have never been involved in any particular sport at a professional level. Therefore, it is not possible to realistically assess their predisposition to high sports results. Probably, it would be necessary to form a comparison group of people who were engaged in this kind of sport but did not achieve any significant results in it, for example, did not acquire a rank of Candidate Master of Sports. The logic of the majority of studies implies that there are alleles (genotypes) that improve the speed and strength qualities of a person, while the opposite genetic variants enhance aerobic qualities. In other words, it is assumed that there is a genotype of an outstanding sprinter and its opposite—the genotype of an outstanding long-distance runner. Certain sports specializations that require a person to simultaneously demonstrate high speed, strength, and aerobic qualities (multisport competition—multiathlon, complex coordination sports, martial arts) do not find their place in such an approach. Due to the significant variety of sports, sports disciplines, and sports roles, it is necessary to assess the possibilities of sports genomics to create sets of genetic markers that increase the chances of a particular individual to achieve high sports results in the chosen sport.

The purpose of the present review is to analyze the prognostic utility of the data presented in the literature on molecular genetic markers of genetic predisposition to achieve outstanding sports results using the example of the C > T variant *of ACTN3 (rs1815739).*

## Materials and methods

### Search strategy and inclusion/exclusion criteria

The search for articles was conducted in accordance with the Preferred Reporting Items for Systematic reviews and Meta-Analysis (PRISMA) guidelines [18]. A search of publications for the analysis was carried out in the databases PubMed and Google Scholar for the keywords ACTN3, sport genetics, athletes, SNP, and sport selection. Full-text articles that met the goals of the study were used for the analysis. A total of 42 studies were included in the analysis [8, 10, 12, 19–57], with a total number of 41,054 individuals (of which 10,442 were in the athlete group and 30,612 in the control group). For each study included in the analysis, the agreement of genotype frequencies with Hardy–Weinberg equilibrium (HWE) was tested, as well as the presence of an excess or deficit of heterozygotes. The *p*_mid_ values were calculated, i.e., exact *p*-values adjusted for the conservativeness of the exact criteria [58], using an online software (https://www.cog-genomics.org/software/stats). To test the presence of an excess or deficiency of heterozygotes, the corresponding exact *p*-values were calculated using the GENEPOP software [59].

It is known that *p*-values do not say anything about the probability of the absence of the effect (about the probability of the null hypothesis), or about the sign of the effect, or about its size. Therefore, interval estimation of the effect size is more informative and has long become a mandatory procedure in statistical analysis. One of the main measures of the deviation of the observed frequencies of genotypes from the HWE is the fixation index *F*_IS_ (inbreeding coefficient). Therefore, to test the overall agreement of genotype frequencies with HWE, the confidence intervals (CIs) for *F*_IS_ were calculated and checked whether they covered the equilibrium value of *F*_*IS*_ = 0 or not. To test the agreement of the frequency of each of the genotypes with the expected one in HWE, the CIs for the observed frequencies were calculated and checked whether they covered the values expected in HWE or not. To test the equality of frequencies of genotypes or alleles, the CI for the frequency difference *D* was also calculated and checked whether they covered the indifferent value *D* = 0 or not. The MetaGenyo (https://metagenyo.genyo.es/) software was used for meta-analysis [60]. Prediction intervals for the overall effect size (OR—odds ratio) was estimated using the Meta-Essentials software (https://www.erim.eur.nl/research-support/meta-essentials/) [61] and/or CMA Prediction interval (https://meta-analysis-workshops.com/pages/predictionintervals).

## Results and discussion

**Fig. 1:**
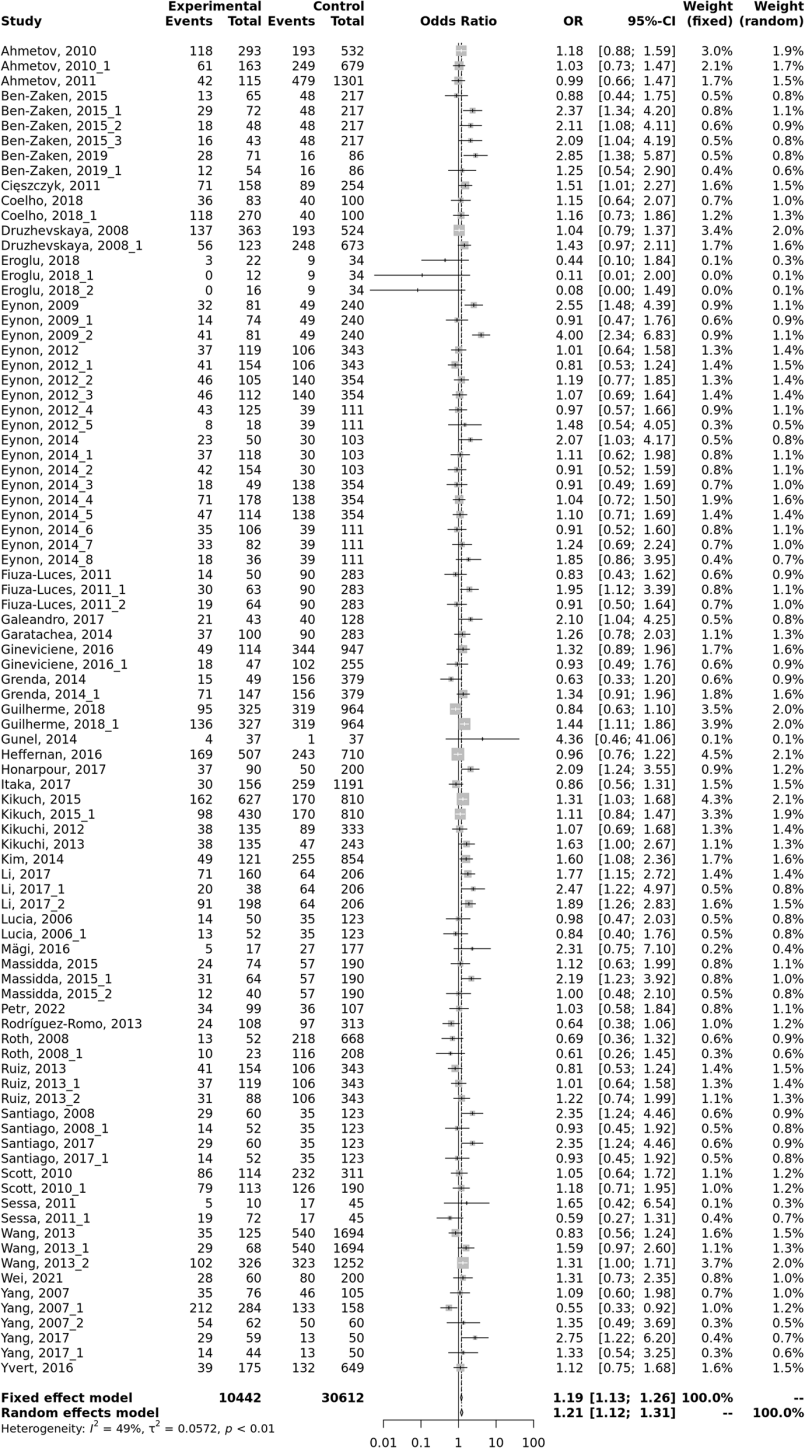
Results of the meta-analysis for the recessive model

In 17 subgroups of athletes, a significant difference *F*_IS_ from zero was found, positive or negative suggesting inbreeding and outbreeding, respectively, as well as a very wide 95% CI for *F*_IS_. Similar results were found for nine control groups. One of the main reasons of this fact could be the unavoidable genotyping errors. For 21 comparisons between a control group and a group of athletes, the difference in genotype proportions was significantly different from zero for at least one genotype. For all other cases, the differences between the frequencies of genotypes were statistically insignificant. The results of a meta-analysis conducted for dominant, codominant, and recessive inheritance models are presented in Fig. 1. The obtained ORs and their 95% CIs are in the range of almost negligible values or have very wide CIs. Thus, no sports or sports roles were found for which the C > T variant of the *ACTN3* gene would be a reliable prognostic marker for assessing an individual predisposition to achieve high sports performance. The results support the conclusion that neutral gene polymorphism—from evolutionary or adaptive point of view—is not a trait that can be selected or used as a predictive tool in sports [62]. To date, most of the associations identified have not proven their practical value [63, 64]. The practical inadequacy of using individual genes to predict sports giftedness at the individual level is based on the following limitations: genotyped variants are not functionally significant and demonstrate incomplete linkage with other significant gene variants; low statistical power of studies, lack of population stratification; heterogeneity of the phenotypes and loci under study. As has been previously shown, the use of a genetic marker to test the phenotypic manifestation of a binary trait (healthy-sick, athlete-non-athlete) depends on the frequency of occurrence of this genotype (allele, haplotype) and the frequency of manifestation of the phenotype under study [65]. If OR < 2.2, then at any frequency of occurrence of this marker, it does not have any diagnostic value. For OR > 5.4 and with a population frequency above 0.3, the marker can be recognized as suitable for mass screenings and professional selection [65]. But such genetic markers have not been found yet and are unlikely to ever be detected.

Contradictory research results, as well as the lack of evidence on their real practical value for the search, selection, and further orientation of young athletes in the choice of a training program, led to a joint statement issued by leading scientists in the field of sports genetics in 2016: to date, there are no scientifically substantiated grounds to believe that the studied molecular genetic markers have a predictive power for the selection of talented athletes as well as for individualization of the training process; test systems based on the results of these studies are misleading and should not be used for these purposes [65]. Without a doubt, the complex of morphofunctional and psychophysiological traits inherent in high-level athletes is based on a variety of genes [66]. However, the mechanisms that determine these links still remain practically unknown. With regard to persons who have not reached the adulthood, the following questions should be asked and resolved: whether sports clubs, sections, and state institutions can require students to provide data on individual genetic characteristics; whether it is possible to deny a young athlete the right to be engaged in a particular sport on the basis of genetic data; who may have an access to genetic data of a minor; what is the mechanism for protecting a child from discrimination for genetic reasons; what consequences may occur for an athlete if they refuse to undergo genetic testing [66–68]. It should also be recognized that with the practical use of such genetic testing, a high risk of obtaining both false-positive and false-negative results and conclusions is inevitable [67]. Such an approach may be acceptable at the population level but cannot be used for individual assessment. “Currently, the predictive ability of sports genetics is zero. There is no direct evidence for the existence of genetic measures of athlete success. The success of an athlete depends primarily on socioeconomic, cultural, and environmental factors. So, the stopwatch is much more useful at predicting a runner’s athletic performance than all those genetics.” Yannis Pitsiladis [69].

The assumption that talent is a fixed capacity that can be identified early, the influence of talent beliefs on athlete development, the different levels of risk for talent selection decisions, biases evident in approaches to athlete selection, the inadequacy of current statistical approaches, the problems with using current performance to predict future outcomes, and how short-term priorities and competition between sports for talented athletes undermine the overall efficiency of athlete development systems [69]. One of the most important issues in the prediction of success in sports based on assumed individual genetic predisposition is uncertainty measured with the prediction intervals [70]. The commonly used confidence interval is an index of precision, not an index of dispersion. It tells us how precisely we have estimated the effect size. It says nothing about how much the effect size varies. The prediction interval (*PI*) reflects the dispersion in effects. In the discussed example of the *C* > *T* variant in the *ACTN3* gene 95% *PI* for the *OR* in a recessive model lies between 0.74 and 1.92. It covers the indifferent value *OR* = 1 and statistically does not differ from zero and tells us that in some 95% of studies comparable to those in the analysis, the true effect size will fall in this interval. PIs for other models are presented in Table 1.

**Table 1:** Summary of the results of the meta-analysis

Recessive model: *CC* vs *CT* + *TT*					
**Model**	**OR**	**95%-CI**	**95% PI**		**Adjusted *p*-value**
Fixed effect	1.19	[1.13; 1.26]	[0.74; 1.92]		10^–10^
Random effect	1.21	[1.12; 1.31]	[0.75; 1.96]		6∙10^–6^
Heterogeneity and publication bias tests:					
***τ***^**2**^	***H***	***I***^**2**^	***Q***	***p*-value**	**Egger’s test *p*-val**
0.06	1.39	0.49	171.0	< 0.001	0.51
**Dominant model: *CC* + *CT* vs *TT***					
**Model**	**OR**	**95%-CI**	**95% PI**		**Adjusted *p*-value**
Fixed effect	1.09	[1.02; 1.17]	[0.57; 2.09]		0.052
Random effect	1.10	[1.02; 1.25]	[0.58; 2.18]		0.17
Heterogeneity and publication bias tests:					
***τ***^**2**^	***H***	***I***^**2**^	***Q***	***p*-value**	**Egger’s test *p*-val**
0.11	1.45	0.52	182.7	< 0.001	0.067
**Overdominant model: *CT* vs *CC* + *TT***					
**Model**	**OR**	**95%-CI**	**95% PI**		**Adjusted *p*-value**
Fixed effect	0.93	[0.89; 0.97]	[0.54; 1.51]		0.016
Random effect	0.90	[0.84; 0.97]	[0.56; 1.54]		0.059
Heterogeneity and publication bias tests:					
***τ***^**2**^	***H***	***I***^**2**^	***Q***	***p*-value**	**Egger’s test *p*-val**
0.06	1.49	0.55	195.2	< 0.001	0.16

Thus, the prediction ability of this genetic marker is useless (despite the high statistical significance of the obtained OR values and the absence of the publication bias). Such conclusion is obviously true for most other genetic markers used in sports genetics. “Very few meta-analyses report prediction intervals and hence are prone to missing the impact of between-study heterogeneity on the overall conclusions. The widespread misinterpretation of random effect meta-analyses could mean that potentially harmful treatments, or those lacking a sufficient evidence base, are being used in practice. Authors, reviewers, and editors should be aware of the importance of prediction intervals” [71]. And the prediction interval should be reported as a part of any meta-analysis where it can be estimated reliably [72].

## Conclusions

Despite numerous attempts to identify genetic variants associated with success in elite sports, progress in this direction remains insignificant. Commercial institutions that provide services in the field of genetic predisposition to the general public in the overwhelming majority of cases do not consider themselves obliged to comply with international bioethical standards for the use and protection of the data they receive. Often, such entities share the data obtained with the third parties (scientific groups or other organizations) and use the collected data for purposes not specified in informed consents [73]. The question of the advantages of genetic testing over the procedures of standard pedagogical and anthropometric testing also remains open. It should be kept in mind that a particular phenotype can be the product of completely different genotypes and even genomes. This is supported by the phenomenon of so-called doubles—unrelated people, sometimes living on different continents, having a striking similarity. This example illustrates the complexity of the task of guessing or predicting the phenotypic manifestation of a particular genome genotype (penetrance), even if we are talking about rare alleles that have a pronounced effect on the phenotype. For example, the carriage of rare highly penetrant pathogenic alleles that cause the development of childhood monogenic diseases does not always lead to the development of the disease. The study of more than half a million genomes made it possible to identify 13 adults who were carriers of eight rare pathogenic variants but did not manifest the disease in them [74].

Even if it were possible to carry out genetic testing to select the most predisposed and perspective individuals, should we do so? The conventional wisdom regarding the use of genetic testing in sports is that it is not acceptable to use such tests before the age of 18 [63, 73]. For those under the age of 18, the following questions must be addressed: whether sports clubs, sections, and government agencies can require students to providing data on individual genetic characteristics; whether it is possible to deny a young athlete the right to engage in a particular sport on the basis of genetic data; who can have access to data on the genetic parameters of the minor; what is the mechanism for protecting the child from discrimination on genetic grounds; what consequences may occur for an athlete if they refuse to undergo genetic testing [66]. The development of an appropriate regulatory framework and control over its implementation by supervisory authorities is becoming vitally necessary. The development of modern technologies in the field of genomics—highly effective sequencing, big data analysis, the use of artificial intelligence, and genome editing—should contribute to the emergence of personalized medicine and gene therapy tools as part of everyday practices. However, these newly arising opportunities pose a number of ethical, moral, social, and personal questions to society. The field of genomics of motor activity is also under the influence of developing genomic technologies, which makes it urgent for the scientific community to formulate common principles and approaches to the procedures of genetic testing of athletes. Modern technical means for obtaining genetic data and the speed of their accumulation significantly surpass our current capabilities for their interpretation and correct application.

## Data Availability

The datasets used and/or analyzed during the current study are available from the corresponding author upon reasonable request.

## Abbreviations

ACE: Angiotensin-I-converting enzyme
ACTN3: Alpha-actinin 3
CI: Confidence interval
CMA: Comprehensive Meta-Analysis
D: Frequency difference
*F*_IS_: Fixation index (inbreeding coefficient)
HWE: Hardy-Weinberg equilibrium
OR: Odds ratio
PI: Prediction interval
SNP: Single-nucleotide polymorphism
SNV: Single-nucleotide variant
